# Vaccine hesitancy about the HPV vaccine among French young women and their parents: a telephone survey

**DOI:** 10.1186/s12889-023-15334-2

**Published:** 2023-04-01

**Authors:** Fatima Gauna, Pierre Verger, Lisa Fressard, Marie Jardin, Jeremy K. Ward, Patrick Peretti-Watel

**Affiliations:** 1 ORS PACA, Observatoire Régional de la Santé Provence-Alpes-Côte d’Azur, Marseille, France; 2 Aix Marseille Univ, IRD, AP-HM, SSA, VITROME, Marseille, France; 3 IHU-Méditerranée Infection, Marseille, France; 4 CERMES3 (INSERM, CNRS, EHESS, Université de Paris), Villejuif, France

**Keywords:** HPV vaccination, Attitude to health, Vaccine hesitancy

## Abstract

**Background:**

The human papillomavirus (HPV) vaccine reduces the burden of cervical and other cancers. In numerous countries, a slow uptakeof this vaccine persists, calling for a better understanding of the structural factors leading to vaccine acceptation. We aimed to assess the attitudes toward HPV vaccination among its intended public to explore its specific characteristics.

**Methods:**

A random cross-sectional telephone survey of the French general population provided data from a sample of 2426 respondents of the target public: the parents of young women and the young women aged 15-25 themselves. We applied cluster analysis to identify contrasting attitudinal profiles, and logistic regressions with a model averaging method to investigate and rank the factors associated with these profiles.

**Results:**

A third of the respondents had never heard of HPV. However, most of the respondents who had heard of it agreed that it is a severe (93.8%) and frequent (65.1%) infection. Overall, 72.3% of them considered the HPV vaccine to be effective, but 54% had concerns about its side effects. We identified four contrasting profiles based on their perceptions of this vaccine: informed supporters, objectors, uninformed supporters, and those who were uncertain. In multivariate analysis, these attitudinal clusters were the strongest predictors of HPV vaccine uptake, followed by attitudes toward vaccination in general.

**Conclusions:**

Tailored information campaigns and programs should address the specific and contrasted concerns about HPV vaccination of both young women and of their parents.

## Introduction

The links between Human Papilloma Virus (HPV) infections and some forms of cancer have been widely reported in the literature [1–4]. Several high-risk types of HPV are key risk factors for cancers in adults including female genital cancers (i.e., cervical, vulvar and vaginal cancer), as well as anal cancers and head and neck cancers in men and women [1, 3]. The primary HPV-related cancer is cervical cancer [5]. HPV types 16 and 18, which cause precancerous lesions and genital cancers, have been found in 71% of cervical cancers [5].

HPV vaccination is a strategic component of the battle to prevent cervical cancers caused by this virus. The World Health Organization (WHO) recommends HPV vaccination combined with screening and education strategies to reduce the impact of these infections on global public health [4–11]. Its implementation appeared to have promise as a means of reducing the burden of cancer. Unfortunately, as of 2019, most estimates showed vaccination coverage that included the last dose below 75% in most countries [11]. HPV vaccination rates continue to be suboptimal in many countries including France, where this vaccine coverage is among the worst in Europe. Only 40.7% of 15-year-old girls born in 2005 received a first dose of HPV vaccine [10–14]. To achieve optimal vaccination rates, continued efforts are needed to better understand the factors associated with attitudes toward this vaccination [4, 5, 9].

Over the past decade, HPV vaccination may have been affected by the rise of vaccine hesitancy (VH) in Europe [15]. In March 2012, the SAGE Working Group on this topic convened to reach a definition of the term, specifying that it is a delay in acceptance or refusal of some vaccines despite their availability [16]. According to the SAGE group, VH results from the combination of lack of confidence, complacency, and convenience issues [16]. VH is context- and vaccine-specific, rather than driven only by a general attitude toward vaccination [17]. Various approaches have been proposed to understand factors predictive of vaccination as well as to map the determinants of VH [11, 18–21]. Factors associated with VH such as lack of trust in health authorities, vaccine-hesitant doctors, and perceived “newness” of vaccines also play a role in HPV vaccination [15, 22, 23]. The literature suggests the existence of different VH clusters and social differentiation between them, varying with the type of belief, vaccine, and country [24].

A considerable amount of literature has examined attitudes toward HPV infection and HPV vaccination, drawing mainly on surveys among parents [25–27] and young women [19, 28]. Two complementary systematic literature reviews have summarized the factors influencing HPV knowledge and vaccine acceptance among young women and their parents and VH in Europe [15, 29]. The literature about VH related to HPV vaccination discusses the most prevalent concerns linked to HPV vaccine uptake; these include but are not limited to insufficient and inadequate information about HPV vaccination, beliefs that the vaccine causes long-term side effects, perceived effectiveness and perceived low risk of HPV/cervical cancer [15, 26]. Here, we address hesitancy toward HPV vaccination by examining the case of France, where this vaccine coverage is among the worst in Europe. Public health authorities in France have recommended HPV vaccination since 2007. Initially intended for girls aged 14–21, then the recommendation was extended for all girls aged 11–14 years old in 2013, when the French High Council for Public Health issued new guidelines. The vaccination schedule requires two to three doses spaced out between six months depending on the vaccine chosen (Gardasil® or Cervarix®) and the girl's age. Prescribed by a general practitioner, these vaccines are reimbursed at 65% by the Health Insurance. Despite the communication efforts of the health authorities, a 2016 national survey found that more than half the French parents of adolescent girls had negative attitudes toward the HPV vaccine or were uncertain of its benefits [30].

The available research on HPV vaccination has identified several barriers to HPV vaccination but tended to oppose those in favor of this vaccine and those opposed to it instead of considering the various forms of reluctance. We explore this diversity and analyse whether there are socially differentiated clusters of VH toward HPV vaccination and how they influence vaccination behavior. Our aims were: 1) to explore hesitancy toward this vaccine among the young women (targeted group) and their parents (who often take the decision) specifically by considering simultaneously four different perceptions related to the disease's severity and its frequency, and the vaccine's efficacy and side effects respectively, as well as their possible combinations; 2) study the potential sociodemographic differences between profiles associated with the different types of vaccine hesitancy; 3) to test the extent to which these profiles to predict self-reported vaccination behavior.

## Methods

### Study setting and participants' characteristics

We used data from the 2016 Baromètre Santé, a national cross-sectional telephone survey addressing health issues in a representative population sample, conducted by the French Public Health Agency (Santé Publique France) [12, 30]. Data collection used a computer-assisted telephone interview (CATI) survey that took place between January and July 2016 in mainland France. It used an overlapping dual-frame design of landline and mobile phone numbers, generated randomly from the prefixes allocated by the electronic communications regulatory authority. All households with at least one French-speaking individual aged 15–75 years were eligible. Among other health-related issues, the 2016 questionnaire dealt with HPV vaccination and the corresponding section targeted two specific categories: on the one hand, parents of at least one girl aged 11–19 years, as it is the intended age category for HPV vaccination in France at the time of survey, and, on the other hand, young women aged 15–25, who were supposed to have had access to the vaccine since it was introduced in France in 2007. One respondent from each household was selected at random for each landline phone or from eligible mobile phone users. The French national commission for computer data and individual freedom (CNIL) approved the survey.

## Measures

Respondents were asked about their attitude toward vaccination in general (from "very favorable" to "not at all favorable"). To capture different structural factors involved in the attitude toward HPV vaccine, we measured reported knowledge and perceptions about this vaccine. Therefore participants were asked whether or not they had ever heard of HPV vaccination and then to either agree or disagree (from “Absolutely” to "Not at all”) with four assertions related to, respectively, the severity and the frequency of HPV infections and the effectiveness and potential side effects of the vaccines against it. These questions were also asked of participants who stated that they had not heard of it, to see the extent to which people may endorse attitudes toward an unknown vaccine driven by their attitude toward vaccination in general. The questionnaire also collected data on HPV vaccine uptake: parents reported their daughters' vaccination status, and young women reported their own. Thus, the HPV vaccine uptake was evaluated by the answers "yes/no/don’t know" of both young women and parents of teenage girls.

Finally, the questionnaire collected information about participants’ sociodemographic background: gender, age, educational level, and household income. The equivalized household income per month was computed taking into account household size and composition, to estimate participants’ standard of living [31].

### Statistical analysis

Data were weighted so the distribution of the main sociodemographic characteristics (gender, age, educational level, geographical region, and urbanization level) matched the sample to the national census. Weights were applied to all statistics.

First, we analyzed the perceptions of HPV infection and vaccination simultaneously, by conducting a cluster analysis to summarize the variety of perceptions reported by participants into contrasting attitudinal clusters toward the HPV vaccination. Items measuring agreement were coded from 1 (“Absolutely”) to 4 ("Not at all”). These scores were transformed into Z-scores before clustering with the standard agglomerative hierarchical procedure [32].

We also investigated the sociodemographic composition of the resulting clusters, as well as their association with attitudes toward vaccination in general, by using χ^2^ independence tests. Then we examined the factors associated with HPV vaccination status, including sociodemographic indicators, the clusters, and attitude toward vaccination in general. Using a logistic model, we used a multimodel averaging approach based on the Akaike information criterion to rank the explanatory variables by their relative importance. This approach estimates all possible models, given the explanatory variables introduced, and computes the final model as the weighted average of all parameters and standard errors from all possible models [33]. We used partial Nagelkerke’s R squares [34] to quantify the partial contributions of each explanatory variable to the dependent variable [35] and relative weights (values between 0 and 1) to classify the explanatory factors according to the level of the evidence of an actual relation to the dependent variable. The explanatory factors were classified as follows: [0–0.5[= no evidence; [0.5–0.75[= weak evidence; [0.75–0.90[= positive evidence; [0.95–0.99[= strong evidence; [0.99–1 [= very strong evidence [36, 37].

## Results

The sample of the 2016 Baromètre Santé included 15,216 respondents with full interviews (participation rate: 50%). The questions about HPV vaccination concerned 2168 participants from two subgroups —young women (45%, mean = 20 years old; SD = 3) and parents of young women (55%, mean = 45 years old; SD = 6) whose attitudes and behavior toward HPV vaccination we sought to study. Among our participants, the overall HPV self-reported vaccine uptake rate was 35.2% (45.8% for young women aged 15–25 years, and 26.6% among parents who reported the vaccination status of a daughter aged 11–19 years.

### Clusters of attitudes toward HPV vaccination

A third (35.1%) of respondents had not heard of HPV vaccines at the time of research (see Table 1). Most respondents nonetheless agreed that HPV infections are severe (93.8%) and frequent (65.1%). Furthermore, 72.3% of respondents considered the HPV vaccine to be effective, although half (54%) reported that it may also cause side effects.

**Table 1:** Attitudes toward HPV vaccination in France, 2016 (cluster analysis, *N* = 2,168)

	Whole sample	Cluster 1 (40.6%) Informed supporters	Cluster 2 (23.6%) Objectors	Cluster 3 (29.2%) Uninformed supporters	Cluster 4 (6.6%) Uncertain	
Opinions toward the HPV vaccine :	% in column					*p*-value*
Have heard of the HPV vaccine:	64.8	100	88.2	0.5	49.4	<0.0001
-Yes	35.1	0	0	99.5	50.6	
- No						
HPV infections are severe:	93.8	95.5	92.9	95.9	77.6	<0.0001
-	5	4.5	7.1	4.1	4.6	
-Absolutely/Somewhat	1.2	0	0	0	17.7	
-Not really/Not at all						
-Don't know						
HPV infections are frequent:	65.1	79.3	46.1	67.4	35.9	<0.0001
-	32	20.5	53.8	32.1	24.5	
-Absolutely/Somewhat	2.9	0.1	0.1	0.5	39.5	
-Not really/Not at all						
-Don't know						
The HPV vaccine is effective:	72.3	97.7	23.5	86.3	30.4	<0.0001
-	24	2.3	76.2	13.7	15.9	
-Absolutely/Somewhat	3.7	0	0.3	0	53.7	
-Not really/Not at all						
-Don't know						
The HPV vaccine may cause side effects:	54	37.7	94.2	49.4	31.1	<0.0001
-	42	61.1	5.8	49.7	14.6	
-Absolutely/Somewhat	4.4	1.2	0	0.9	54.3	
-Not really/Not at all						
-Don't know						

^*^ *p*-values from χ^2^ independence tests

The cluster analysis produced four contrasting profiles. Before examining each cluster more closely, two general results must be emphasized. First, despite the gaps in knowledge about HPV vaccination (35% of participants had not heard of this vaccine, from 0.5% in Cluster 3 to 100% in Cluster 1), there was consensus across the four clusters regarding the severity of HPV infections (between 77.6% and 95.9% of participants considered them absolutely or somewhat severe). Second, the four clusters displayed contrasting opinions about the potential side effects of the vaccines, but in each cluster at least one third of respondents believed that HPV vaccines could cause severe side effects. Participants in the different clusters agreed that HPV infections are serious and the vaccine is effective but had divided views about the frequency of these infections and about the safety of the vaccine.

Cluster 1 comprised 40.6% of participants. All of them had heard of HPV vaccines. Nearly all (95.5%) agreed that HPV infections are severe, and 79.3% perceived HPV as frequent. The vast majority considered HPV vaccines to be effective (97.7%), but more than a third were concerned about possible side effects (37%). We labeled this profile as Informed supporters.

Respondents in cluster 2 (23.6% of the sample), were labeled as *Objectors*. Most (88%) had heard of the HPV vaccine, and agreed that HPV infections are severe (92.9%). Among them, only 46.1% agreed that HPV infections are frequent, and almost all were concerned about its potential side effects (94.2%).

We labeled Cluster 3 *Uninformed supporters* (29.2% of the sample) because 99.5% of respondents in this cluster reported they had not heard of the HPV vaccine. Most of them considered HPV infections to be serious (95.9%), and a large majority agreed that these infections are common (67.4%). According to 86.3% of the respondents in this cluster, HPV vaccines are effective but 49.4% thought that they might have side effects.

Finally, in Cluster 4, only half of the participants (who represented 6.6% of the whole sample) had heard of this vaccine. This cluster concentrated most of the "Don’t know" answers, and was labeled as *Uncertain*. Among them, 77.6% agreed HPV infections are severe (17.7% didn’t know) and a third agreed they are frequent (39.5% didn’t know). The questions concerning their views of the effectiveness of the vaccines and their potential to cause side effects showed high levels of uncertainty (respectively 53.7% and 54.3% did not know).

### Characterization of attitudinal clusters toward HPV vaccination

Our results showed that parents were more frequently uncertain toward HPV vaccination than young women (see Table 2). Fathers were overrepresented among uninformed supporters, while mothers, and especially those aged 25–45, were more frequently objectors. On the contrary, younger women (15–19) were more supportive of this vaccination.

**Table 2:** Factors associated with specific attitudes toward the HPV vaccination (France, 2016)

	All	Cluster 1 (40.6%) *Informed supporters*	Cluster 2 (23.6%) *Objectors*	Cluster 3 (29.2%) *Uninformed supporters*	Cluster 4 (6.6%) *Uncertain*	
	% in columns					*p*-value^*^
Gender/age						< 0.0001
Young women						
15–19 years old	23.3	24.5	20.6	28.3	3.5	
20–25 years old	21.4	26.4	22.8	15.7	10.5	
Parents of young women						
Women						
26–45 years old	21.2	19.7	27.6	16.3	29.8	
> 45 years old	13.3	13.2	16.1	9.2	22.2	
Men						
25–45 years old	8.6	6.8	4.9	13.4	12.4	
> 45 years old	12.1	9.4	8	17.1	21.5	
Educational level:						< 0.0001
- < high-school	42.8	30.6	41.4	57.8	56.7	
- high-school	27.1	32.4	26.3	23.6	12.1	
- up to 3 years completed at university	19.6	23	23.5	11.6	19	
- > 3 years completed at university	10.5	13.9	8.8	6.9	12.1	
Household income						< 0.0001
1-Low	44.2	38.3	44.7	52	52.4	
2-Medium	28.9	31	28.4	25.8	32.1	
3-High	20.4	23.7	22.6	14.8	17.7	
4- No answer	6.4	7.1	4.3	7.4	5.2	
Favorable to vaccination:						< 0.0001
-Strongly/Somewhat	79.0	87.6	58.8	84.8	73.2	
-Not really/Not at all	21.0	12.4	41.2	15.2	26.8	

^*^ *p*-value by χ^2^ independence tests

Educational level was also strongly correlated with these attitudinal clusters. Objectors had an educational profile close to the average, while informed supporters were more educated (69.4% had completed high-school vs 42.2% to 58.6% in other clusters). Uninformed supporters and uncertain participants were the least educated.

Results for household income were similar: objectors had an average profile for household income per consumption unit, while informed supporters were wealthier and low-income households were overrepresented among the two other clusters.

Finally, the majority of objectors were nonetheless favorable to vaccination in general (58.8%), versus 87.6% of informed supporters, 84.8% of uninformed supporters, and 73.2% among the uncertain.

### Factors associated with HPV vaccine reported uptake

In the bivariate analyses, self-reported HPV vaccine uptake was significantly more frequent among informed supporters (52.7%, versus 17.6% to 29.2% for other clusters) and young women aged 20–25 (52.9%) (see Table 3). This coverage was also lower among both the lowest and the highest educational level categories, while it was weakly correlated with household income level. Finally, HPV vaccination coverage was twice as higher among participants who supported vaccination in general than among those who did not.

**Table 3:** Factors associated with HPV vaccination status, bivariate analysis (France, 2016)

	Yes *n* = 848	No *n* = 1473	Don't know *n* = 86	
				*p*-value^a*^
Clusters				< 0.001
Cluster 1 (*informed supporters*)	52.7	44.1	3.2	
Cluster 2 (*objectors*)	23.6	85.6	1.6	
Cluster 3 (*uninformed supporters*)	29.2	62.2	4.7	
Cluster 4 (*uncertain*)	17.6	74.1	8.3	
Gender/age				< 0.001
Young women				
15–19 years old	39.4	54.2	6.4	
20–25 years old	52.9	46	1.1	
Parents of young women				
Women				
26–45 years old	25.8	73.2	1.0	
> 46 years old	30.5	67.5	2.0	
Men				
25–45 years old	20.5	73.5	6.1	
> 45 years old	27.8	65.4	6.8	
Educational level				< 0.001
< High-school	30.2	63.9	5.9	
High-school	42.2	56.1	1.7	
1–3 years completed at university	37.9	60.2	1.9	
> 3 years completed at university	32.8	65.2	2.0	
Household Income				0.02
Low (*n* = 1073)	36.3	58.8	4.9	
Medium (*n* = 702)	32.3	65.7	2.0	
High (*n* = 496)	35.5	62.1	2.4	
no answer	40.6	54.4	5.0	
Favorable to vaccination in general:				< 0.001
Absolutely/Somewhat	39.6	56.7	3.7	
Not really/Not at all	18.6	78.3	3.1	

^*^ *p*-value resulting from χ^2^ independence tests

The multimodel averaging approach showed that informed supporters, young women in the 20–25 year-old age range, and participants who were favorable to vaccination in general were most likely to report HPV vaccination, and the corresponding three variables obtained the highest importance weight in our model (very strong) (Table 4). Once controlled for attitudinal profiles, we found evidence of only a weak association between educational level and HPV vaccination status and no evidence of a significant effect concerning household income.

**Table 4:** Factors associated with HPV vaccine uptake, multimodel averaging approach (France, 2016)

	Partial R^2^	aOR#[95% CI]	Weight*	Evidence	Rank
Clusters	0.13				
Cluster 1		1	1.000	Very strong	1
Cluster 2		0.15 [0.11;0.20]			
Cluster 3		0.49 [0.40;0.61]			
Cluster 4		0.27 [0.17;0.42]			
Gender/age			1.000	Very strong	2
Young women					
15–19 years old		1			
20–25 years old		1.98 [1.50;2.61]			
Parents of young women:					
Women	0.07				
26–45 years old		0.60 [0.45;0.80]			
> 46 years old		0.79 [0.57;1.09]			
Men					
25–45 years old		0.41 [0.27;0.62]			
> 45 years old		0.65 [0.46;0.92]			
Favorable to vaccination in general:			1.000	Very strong	3
Absolutely/Somewhat	0.02	1			
Not really/Not at all		2.27 [1.73;2.96]			
Educational level			0.580	Weak	4
< High-school	0.00	1			
High-school		1.18 [0.94;1.49]			
1–3 years completed at university		1.09 [0.83;1.42]			
> 3 years completed at university		0.76 [0.54;1.07]			
Household Income	0.00		0.190	None	5
Low		1			
Medium		0.83 [0.66;1.04]			
High		0.98 [0.75;1.28]			
no answer		0.89 [0.61;1.31]			

^*^According to Viallefont’s classification [0–0.5]: no evidence; [0.5–0.75]: weak evidence; [0.75–0.90]: positive evidence; [0.95–0.99]: strong evidence; [0.99–1]: very strong evidence

^#^ adjusted odds ratio

## Discussion

### Main results

In our study, 35.2% of participants reported HPV vaccine uptake. This result was reasonably close to the actual French national coverage [14]. Combining opinions on the frequency and severity of HPV infections, and HPV vaccination efficacy and side effects, we found four contrasting profiles of attitudes toward this vaccination (informed supporters, objectors, uninformed supporters, and uncertain) among young women and parents of young women. Each profile contained a substantial proportion of participants concerned about potential side effects of the vaccine. These profiles differ mainly according to reported knowledge and perceptions of the risk–benefit of vaccination. Informed supporters reported to be informed about the HPV vaccine and considered the vaccine to be effective even though some of them were unsure about the safety of the vaccines. In contrast, Objectors, although they reported to be globally informed about the vaccine, considered the disease rather rare and the vaccination not necessarily effective or safe. The other two profiles are characterized by a low reported knowledge of HPV vaccine. However, the Uninformed supporters considered it effective but didn’t have a shared perception about its safety. The last group identified, Uncertain, grouped respondents reporting uncertainty about their perceptions of the vaccine. These profiles were also significantly correlated with participants’ sociodemographic background. In multivariate analysis, these attitudinal clusters were the strongest predictors of HPV vaccine uptake, but attitudes toward vaccination in general also predicted uptake strongly.

### Study limitations

Before discussing our results, we must acknowledge several limitations of our study. First, this study shares the usual shortcomings of quantitative telephone surveys, including a moderate participation rate (50%). The announcement letter describing the survey and requesting participation did not give any details about the topics to be investigated: thus there is no reason to suspect that respondents’ answers regarding the attitudes toward the HPV vaccine and the vaccine uptake were correlated with non-participation. In addition, the data were weighted for various factors that are known to often be associated with survey participation.

Second, like any data collection based on self-report, this survey is subject to social desirability and recall biases, especially regarding past vaccination of participants’ daughters.

### Attitudes toward HPV vaccination

It has been frequently claimed that contemporary VH has been fueled by the very success of vaccination in controlling and eliminating diseases: severe infections that were previously common have almost disappeared, and so people stopped worrying about them (e.g., André, 2003 [38]). In our study, however, young women and their parents were aware of the frequency and severity of HPV infections. This certainly does not mean there are no information problems, as more than a third of respondents reported that they had never heard of HPV vaccines. Information issues about them have already been identified as the main barrier to this vaccination [15, 18, 29] and consequently as a key lever for improving it [39]. The other major barrier to HPV vaccination highlighted by previous studies involves concerns about vaccine safety [18, 29]. These worries were shared by half of respondents and were quite pervasive, being present in each of the four contrasting attitudinal profiles toward HPV vaccination, including informed supporters, in whom at least one third had such concerns. Our multivariate analysis also echoed these results as the objectors (nearly all of whom considered that the HPV vaccine might cause adverse side effects) and the uncertain group were less likely to report HPV vaccination. Many mothers face precisely this dilemma: they know that HPV is dangerous but they remain uncertain about this vaccine's safety.

### HPV vaccination & vaccine hesitancy

The contrasting attitudinal clusters, based on perceptions related to the specific risks and benefits of HPV vaccination, turned out to be slightly more predictive of this vaccination status than attitudes toward vaccination in general. This reflects the specificity of contemporary VH, which is often not guided by a general attitude toward vaccination, but that instead takes the specificities of each vaccine and each context into account [17, 30]. Nevertheless, general attitudes toward vaccination still play a significant role as a determinant of HPV vaccine uptake. A recent study also supported this result, as previous vaccine refusal for a child, which is a good proxy of this general attitude, remained a significant factor in the decision about HPV vaccination, together with awareness of the vaccine's existence [26]. These general attitudes probably capture some aspects related to people’s lack of trust in the health care system and health authorities, which is a systemic issue in contemporary societies and plays an important part in VH [17, 40–42].

### Sociodemographic background and HPV vaccination

Young women were more supportive of HPV vaccination than their parents. Moreover, among young women, the oldest (those aged 20–25 rather than 15–19 years) were more likely to report complete HPV vaccination: this may result from both the mechanical effect of age (older participants have had more opportunities to be vaccinated during their lifetime) and a more supportive attitude toward this vaccination (in line with Patel et al. 2016 [40]). Among parents, fathers were more frequently uncertain or uninformed supporters, which probably reflects the fact that they are usually much less engaged in the vaccination decisions about their children than mothers [43], at least in western cultural contexts where taking care of children’ health is considered a mother’s duty [44]**.** Finally, mothers were more frequently objectors. This gender effect has already been observed for other recent vaccines in France (for the H1N1 vaccine, see [45], for the COVID-19 vaccine, see [46]), and a number of studies conducted in other countries also mentioned a higher hesitancy among women for the COVID-19 vaccine [47–49]. To our knowledge, a wide range of explanations have been put forward ranging from higher tendency for risk aversion, lower trust in medical institutions to a higher likelihood of crossing vaccine-critical information [44, 46]. In the case of HPV vaccination, the campaigns have emphasized its effectiveness in preventing cervical cancer over other HPV-related conditions, leading to errors in the public's risk assessment. In addition, the arguable overlap of science, politics, economics, and beliefs about gender roles that led to the initial focus on women may have had a negative impact on women's confidence in the vaccine [50]. We can hypothesize that women, who are often the bearers of reproductive work that is heavily framed by preventive measures, are more likely to develop critical dispositions that allow them to express concerns about these vaccines and the ability to make choices.

The relation between participants’ socioeconomic status and their attitudes toward vaccination may depend on both the vaccine considered and the national context [22]. Previous studies have found that socioeconomic status and especially educational level are correlated with HPV knowledge [18] and HPV vaccine uptake [27]. In our study, similarly, the informed supporters of HPV vaccination had a higher educational level on average, as well as better household income. Nevertheless, it is worth mentioning that objectors had average profiles for education attainment and living conditions, while the less educated and lower income households were overrepresented among the less informed (uninformed supporters and uncertain). In other words, at least in France, although wealthier and more educated people are more likely to support HPV vaccination, objections to it do not result from ‘poor people’s fears’ fueled by lack of material, social or cognitive resources [43, 45].

## Conclusion

Overall, in 2016, a majority of French people supported HPV vaccination. However, there is still great room for improvement regarding information issues, as a third had never heard of this vaccination, while half shared concerns about its safety. Tailored information campaigns and programs should consider young women and their parents as distinct targets who may have different concerns. Vaccination uptake strongly depends on specific attitudes toward HPV vaccination, which can be enhanced by information campaigns, but also on general attitudes toward vaccination, which involve trust issues.

## Data Availability

Raw data came from the 2016 “Baromètre santé”, a study conducted by the French Public Health Agency (Santé Publique France), available on request from the agency. Moreover, the datasets used and/or analyzed during the current study available from the corresponding author on reasonable request.
